# The Conserved Spore Coat Protein SpoVM Is Largely Dispensable in *Clostridium difficile* Spore Formation

**DOI:** 10.1128/mSphere.00315-17

**Published:** 2017-09-20

**Authors:** John W. Ribis, Priyanka Ravichandran, Emily E. Putnam, Keyan Pishdadian, Aimee Shen

**Affiliations:** a Department of Molecular Biology and Microbiology, Tufts University School of Medicine, Boston, Massachusetts, USA; b Department of Microbiology and Molecular Genetics, University of Vermont, Burlington, Vermont, USA; University of Iowa

**Keywords:** *Clostridium difficile*, SpoIVA, SpoVM, coat, morphogenesis, spore

## Abstract

The spore-forming bacterial pathogen *Clostridium difficile* is a leading cause of health care-associated infections in the United States. In order for this obligate anaerobe to transmit infection, it must form metabolically dormant spores prior to exiting the host. A key step during this process is the assembly of a protective, multilayered proteinaceous coat around the spore. Coat assembly depends on coat morphogenetic proteins recruiting distinct subsets of coat proteins to the developing spore. While 10 coat morphogenetic proteins have been identified in *Bacillus subtilis*, only two of these morphogenetic proteins have homologs in the *Clostridia*: SpoIVA and SpoVM. *C. difficile* SpoIVA is critical for proper coat assembly and functional spore formation, but the requirement for SpoVM during this process was unknown. Here, we show that SpoVM is largely dispensable for *C. difficile* spore formation, in contrast with *B. subtilis*. Loss of *C. difficile* SpoVM resulted in modest decreases (~3-fold) in heat- and chloroform-resistant spore formation, while morphological defects such as coat detachment from the forespore and abnormal cortex thickness were observed in ~30% of *spoVM* mutant cells. Biochemical analyses revealed that *C. difficile* SpoIVA and SpoVM directly interact, similarly to their *B. subtilis* counterparts. However, in contrast with *B. subtilis*, *C. difficile* SpoVM was not essential for SpoIVA to encase the forespore. Since *C. difficile* coat morphogenesis requires SpoIVA-interacting protein L (SipL), which is conserved exclusively in the *Clostridia*, but not the more broadly conserved SpoVM, our results reveal another key difference between *C. difficile* and *B. subtilis* spore assembly pathways.

**IMPORTANCE** The spore-forming obligate anaerobe *Clostridium difficile* is the leading cause of antibiotic-associated diarrheal disease in the United States. When *C. difficile* spores are ingested by susceptible individuals, they germinate within the gut and transform into vegetative, toxin-secreting cells. During infection, *C. difficile* must also induce spore formation to survive exit from the host. Since spore formation is essential for transmission, understanding the basic mechanisms underlying sporulation in *C. difficile* could inform the development of therapeutic strategies targeting spores. In this study, we determine the requirement of the *C. difficile* homolog of SpoVM, a protein that is essential for spore formation in *Bacillus subtilis* due to its regulation of coat and cortex formation. We observed that SpoVM plays a minor role in *C. difficile* spore formation, in contrast with *B. subtilis*, indicating that this protein would not be a good target for inhibiting spore formation.

## Abstract (precis)

The spore-forming obligate anaerobe *Clostridium difficile* is the leading cause of antibiotic-associated diarrheal disease in the United States. When *C. difficile* spores are ingested by susceptible individuals, they germinate within the gut and transform into vegetative, toxin-secreting cells. During infection, *C. difficile* must also induce spore formation to survive exit from the host. Since spore formation is essential for transmission, understanding the basic mechanisms underlying sporulation in *C. difficile* could inform the development of therapeutic strategies targeting spores. In this study, we determine the requirement of the *C. difficile* homolog of SpoVM, a protein that is essential for spore formation in *Bacillus subtilis* due to its regulation of coat and cortex formation. We observed that SpoVM plays a minor role in *C. difficile* spore formation, in contrast with *B. subtilis*, indicating that this protein would not be a good target for inhibiting spore formation.

## INTRODUCTION

The Gram-positive, endospore-forming, gastrointestinal pathogen *Clostridioides difficile* (1, 2), more commonly known as *Clostridium difficile*, is a leading cause of health care-associated infections in the United States (3). *C. difficile-*associated disease ranges from gastroenteritis to more severe conditions like pseudomembranous colitis and toxic megacolon (4). The vegetative form of *C. difficile* causes these disease symptoms through its secretion of inflammation-inducing toxins (4), while its spore form is essential for *C. difficile* to transmit disease because *C. difficile* is an obligate anaerobe (5). Thus, spore formation allows *C. difficile* to survive exit from the host, and spore germination is essential for *C. difficile* to initiate infection (6).

Endospore formation is an ancient, highly coordinated developmental process (7, 8) that generates a metabolically dormant spore from the asymmetric division of a vegetative cell. Asymmetric division creates a larger mother cell and a smaller forespore. The mother cell engulfs the forespore in a process analogous to phagocytosis, leaving the double-membrane bound forespore suspended within the mother cell cytosol. A thick layer of modified peptidoglycan known as the cortex is built upon the germ cell wall present between the two membranes surrounding the forespore. The cortex helps maintain metabolic dormancy and confers heat resistance (9, 10). A series of concentric proteinaceous shells known as the coat is also assembled around the outermost forespore membrane. This external layer functions to protect spores against oxidative, chemical, and enzymatic insults (11) and can protect spores following phagocytosis by eukaryotic organisms (12).

In *Bacillus subtilis*, the coat is made of ~80 different proteins that are assembled in a hierarchal manner around the forespore (11). Landmark proteins recognize the forespore and subsequently recruit additional proteins to create four discrete layers: the basement layer, inner coat, outer coat, and crust (13). The basement layer is the most important of these layers, since mutants deficient in this layer fail to make heat-resistant spores (14–16). A key component of the basement layer is SpoVM (here referred to as VM [14]), a 26-amino-acid small protein (17) that anchors the basement layer to the forespore membrane. VM function depends on its ability to recognize the positive curvature of the forespore and embed itself within the mother cell-derived membrane (18, 19). VM directly binds to and recruits SpoIVA (here referred to as IVA), an ATPase that forms highly stable polymers (20) that encase the forespore (21). This interaction (22) is required for both proteins to preferentially surround the forespore (22–24).

Both IVA and VM are essential morphogenetic proteins in *B. subtilis*, since *IVA* and *VM* mutants fail to properly assemble the coat and make cortex (14, 15). The defect in cortex synthesis presumably underlies their failure to make heat- and chloroform-resistant spores (15, 25, 26). If IVA fails to encase the forespore, a quality control mechanism prevents cortex formation and eventually leads to lysis of the sporulating cell (26). This mechanism is dependent on CmpA (cortex morphogenetic protein A [27]), a ClpXP adaptor protein that targets improperly localized IVA for degradation (26).

IVA also binds to and recruits another coat morphogenetic protein, SpoVID (28) (here referred to as VID). This interaction depends on region A in VID’s C terminus, although it is independent of VID’s C-terminal LysM domain (23). VID then recruits additional coat morphogenetic proteins, SafA and CotE (29–31), that direct assembly of the inner and outer coat layers, respectively (11).

Notably, of the 10 *B. subtilis* coat morphogenetic proteins that have been identified to date (11), only VM and IVA are widely conserved in the *Bacilli* and *Clostridia*, with IVA being strictly conserved (7, 32) and CmpA, VID, SafA, and CotE being conserved exclusively in the *Bacillales* (7, 33). As a result, relatively little is known about spore coat assembly in the *Clostridia*. Preliminary analyses in *C. difficile* of this process have identified SipL as a clostridium-specific functional homolog of VID (34), although unlike *B. subtilis* VID, SipL is essential for heat-resistant spore formation in *C. difficile*. In recombinant coaffinity purification analyses, SipL directly binds to IVA through SipL’s C-terminal LysM domain (34). Given that VID binding to IVA in *B. subtilis* occurs independently of VID’s LysM domain (23), the mechanism by which *C. difficile* IVA recognizes SipL fundamentally differs from how *B. subtilis* IVA recognizes VID.

Interestingly, although *C. difficile* IVA and SipL are dispensable for cortex formation, both coat morphogenetic proteins are required for heat-resistant spore formation (34). These observations contrast with *B. subtilis* (14, 15), where *IVA* mutants fail to make cortex and heat-resistant spores and *VID* mutants make cortex and exhibit modest defects in heat-resistant spore formation. Since these findings are consistent with the absence of CmpA homologs in the *Clostridia* (27, 33), cortex and coat syntheses appear to be less tightly linked in *C. difficile* than they are in *B. subtilis*.

Given that VM is the only other major coat morphogenetic protein that has a homolog in *C. difficile* (7), in this study we sought to determine whether VM was similarly critical for *C. difficile* spore formation using genetic, cytological, and biochemical analyses.

## RESULTS

### Key structural features of VM are conserved between *C. difficile* and *B. subtilis*.

**FIG 1:**
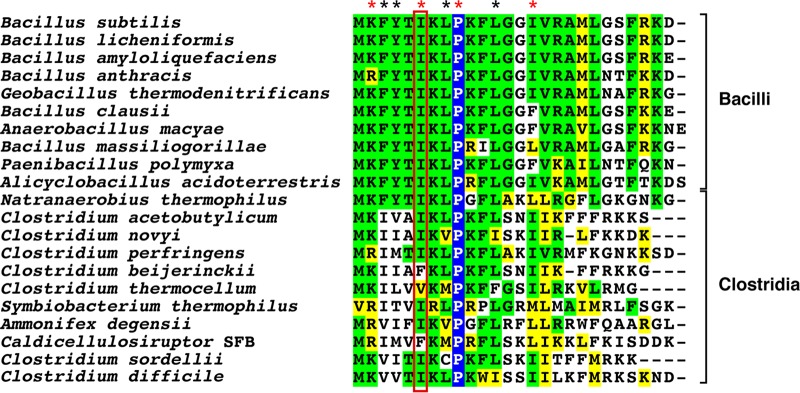
Sequence alignment of VM homologs in the *Bacilli* and *Clostridia*. Pro9 is the only residue that is completely conserved (boxed in blue with white font). This residue introduces a kink into the helical structure of VM (18) and is essential for it to specifically recognize positively curved membranes (19, 24). Conserved identical residues are boxed in green, and conserved similar residues are boxed in yellow. Sequences were derived from the work of Galperin et al. (7). Residues whose mutation to alanine causes severe defects in *B. subtilis* VM function (35) are indicated with an asterisk, while red asterisks highlight residues that have been shown or are presumed to activate the CmpA pathway in *B. subtilis*. K2A and I15A mutations prevent cortex production and activate the CmpA-dependent checkpoint pathway (26, 27), while I6A (highlighted by the red box) prevents binding to IVA and IVA encasement of the forespore (22) and thus presumably also activates CmpA.

Before assessing the requirement for *C. difficile* VM during spore formation, we first compared its primary sequence to *B. subtilis* VM. Extensive mutagenesis analyses have been performed on *B. subtilis* VM (35), and specific functions have been identified for a subset of its 26 residues (22, 26, 27). This sequence alignment revealed that many of the residues previously identified to be critical for *B. subtilis* VM function are conserved in *C. difficile* VM (Fig. 1) (22, 26, 27, 35). Indeed, most of these functionally important residues are conserved across VM homologs in the *Bacilli* and *Clostridia*, suggesting that VM may be under negative selection.

We also analyzed whether *C. difficile VM* is expressed during sporulation and compared its regulation to that of *B. subtilis VM*. Analysis of transcriptome sequencing (RNA-Seq) data previously generated in the JIR8094 strain background (36) revealed that *VM* is transcribed during sporulation under the control of the early-stage mother cell-specific sigma factor σ^E^. This regulation was confirmed using quantitative reverse transcription PCR (qRT-PCR) analyses on a separate set of RNA samples (see Fig. S1B in the supplemental material). This expression pattern differs slightly from *B. subtilis VM*, which for its expression requires SpoIIID (14), a transcription factor that acts downstream of σ^E^ (37). Notably, while σ^E^ controls the transcription of *IVA*, *sipL*, and *VM* in *C. difficile*, *IVA* and *sipL* are expressed at 50- to 100-fold-higher levels than *VM* in the RNA-Seq analyses (Table S3 in Data Set S1).

10.1128/mSphere.00315-17.1 FIG S1  Transcriptional analyses of the *CD25622-VM* locus. (A) Representative image of Integrative Genomics Viewer software (65) used to visualize *CD25622-VM* in RNA-Seq data of sporulating JIR8094 and associated sigma factor mutants from reference 36. The reads mapped to this locus are shown. The direction of transcription is indicated by the angled bracket. (B) qRT-PCR analyses of *VM* transcript levels in wild-type JIR8094, *spo0A* mutant, *sigE* mutant, *spoIIID* mutant, and *sigK* mutant strains relative to *spo0A* mutant. *spoIIID* and *sigK* expression depend on *sigE*, which encodes the mother cell-specific sigma factor σ^E^. ***, *P* < 0.005. Download FIG S1, TIFF file, 0.1 MB. Copyright © 2017 Ribis et al. 2017 Ribis et al. This content is distributed under the terms of the Creative Commons Attribution 4.0 International license.

10.1128/mSphere.00315-17.7 DATA SET S1  Table S1 shows strains and plasmids used in this study. Table S2 shows primers used in this study. Table S3 shows expression levels of select sporulation genes as measured by RNA-Seq. Table S4 shows sporulation efficiency as measured by chloroform resistance. Table S5 shows frequency of forespore morphological abnormalities detected by phase-contrast microscopy. Download DATA SET S1, PDF file, 0.1 MB. Copyright © 2017 Ribis et al. 2017 Ribis et al. This content is distributed under the terms of the Creative Commons Attribution 4.0 International license.

### *C. difficile* VM is largely dispensable for functional spore formation.

**FIG 2:**
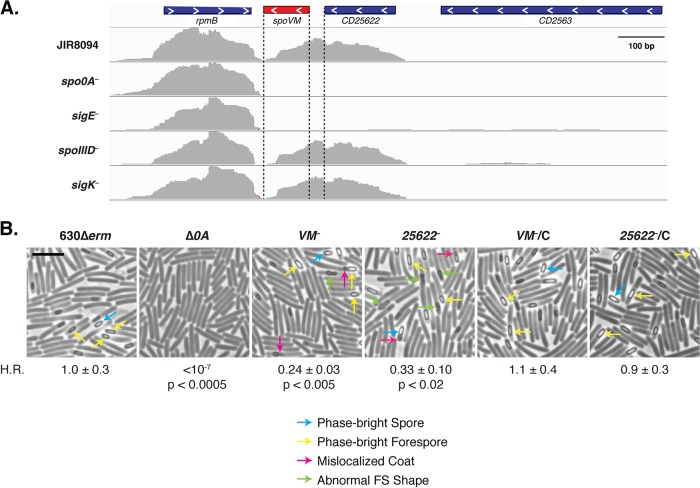
The *CD25622-VM* locus is largely dispensable for heat-resistant spore formation. (A) RNA-Seq transcript data of the *CD25622-VM* locus in wild-type JIR8094 and *spo0A*, *sigE*, *spoIIID*, and *sigK* mutant TargeTron insertion strains (36) visualized using the Integrative Genomics Viewer software (65). *spo0A* encodes the master transcriptional regulator necessary for induction of sporulation. *sigE* encodes σ^E^, a mother cell-specific sigma factor that activates *spoIIID* transcription (59, 66, 67). *spoIIID* encodes a transcription factor that coordinately activates *sigK* transcription along with σ^E^ (36, 67). The proximity of *C. difficile VM* to *rpmB*, which encodes a ribosomal subunit protein, is conserved relative to other *VM*-carrying organisms (7). *CD25622* appears to be unique to the *Peptostreptococcaceae* (41). The angled brackets indicate the direction of transcription. (B) Phase-contrast microscopy of wild-type 630Δ*erm*, Δ*0A*, and *VM* mutant and *CD25622*-TargeTron mutants and their complements (*VM*::*ermB*/C and *25622*::*ermB*/C) sporulating cultures at 20 h. Examples of phase-bright forespores and spores are marked using yellow and blue arrows, respectively. Select forespores with abnormal morphologies are delineated by green arrows, while regions that may correspond to mislocalized coat are highlighted in pink. H.R. refers to the heat resistance of each strain relative to the wild type. The means and standard deviations shown are based on four biological replicates. Statistical significance relative to the wild type was determined using a one-way ANOVA and Tukey’s test. Bars, 5 µm.

Interestingly, during the visualization of the direct reads generated by RNA-Seq we noticed that *VM* is actually cotranscribed downstream of *CD25622* (Fig. 2A and S1B), a gene that encodes a 50-amino-acid protein of unknown function that appears to be primarily conserved among the *Peptostreptococcaceae* family. This raised the possibility that both VM and CD25622 may play important roles during *C. difficile* spore formation. To assess their functional contribution during sporulation, we constructed TargeTron insertions in *VM* and *CD25622* in both the 630Δ*erm*Δ*pyrE* and JIR8094 strain backgrounds (Fig. S2). The 630Δ*erm*Δ*pyrE* strain was used to facilitate chromosomal complementation of the *VM* mutants at the *pyrE* locus using allele-coupled exchange (ACE) (38). Nevertheless, in both strain backgrounds, the TargeTron insertions in *CD25622* likely have polar effects on *VM* expression such that they are effectively *CD25622-VM* double mutants.

10.1128/mSphere.00315-17.2 FIG S2  Construction of *VM*, *CD25622*, and Δ*IVA* mutants. (A) Schematic of the TargeTron-based insertions in *VM* (*VM*::*ermB*) and *CD25622* (*CD25622*::*ermB*). The TargeTron insertion in *CD25622* is predicted to have polar effects on *VM* expression. (B) Colony PCR of *VM*::*ermB* (*VM*−) and *CD25622*::*ermB* (*CD25622*−) mutants in the 630Δ*erm*Δ*pyrE* and JIR8094 strain backgrounds. The group II intron is ~2 kb. (C) Schematic and colony PCR of the *spoIVA* deletion in 630Δ*erm*Δ*pyrE*. Download FIG S2, TIFF file, 0.3 MB. Copyright © 2017 Ribis et al. 2017 Ribis et al. This content is distributed under the terms of the Creative Commons Attribution 4.0 International license.

To assess the functional requirement for VM and CD25622 during sporulation, we compared the heat resistance of sporulating cultures of *VM* mutant and *CD25622* mutant strains to that of the wild type. *C. difficile* 630Δ*erm VM*::*ermB* and *25622*::*ermB* exhibited a relatively modest ~3- to 4-fold heat resistance defect relative to the wild type (*P* < 0.02, Fig. 2B) in contrast with the >6-log defects of *B. subtilis VM* mutants relative to the wild type (25). Importantly, the heat resistance defects of 630Δ*erm VM* mutants could be fully complemented by expressing *CD25622-VM* from the *pyrE* locus (Fig. 2B). Since *VM*::*ermB* and *CD25622*::*ermB* mutants in the JIR8094 background exhibited a similar ~5-fold heat resistance defect relative to the wild type (Fig. S3A), loss of VM leads to minor but reproducible decreases in heat-resistant spore formation. Furthermore, CD25622 would appear to have little impact on *C. difficile* sporulation given that the heat resistance defects of *VM*::*ermB* and *CD25622*::*ermB* mutants in both strain backgrounds were essentially identical.

10.1128/mSphere.00315-17.3 FIG S3  The *CD25622-VM* locus is largely dispensable for heat-resistant spore formation in JIR8094. (A) Phase-contrast microscopy of sporulating cultures of wild-type JIR8094, *spo0A* mutant, and *VM* and *CD25622-VM* TargeTron mutants at 21 h. Examples of phase-bright forespores and spores are marked using yellow and blue arrows, respectively. Forespores with abnormal morphologies are denoted by green arrows, while regions that likely correspond to mislocalized coat are marked in pink. HR refers to the heat resistance of each strain relative to the wild type. The means and standard deviations shown are based on three biological replicates. Statistical significance relative to the wild type was determined using one-way ANOVA and Tukey’s test. Bars, 5 µm. (B) Transmission electron microscopy (TEM) analyses of sporulating cells of wild-type JIR8094 and *VM* and *CD25622-VM* TargeTron mutants and their complements (*VM*^−^/C and *25622*^−^/C). Bars, 500 nm. Blue arrows mark coat layers that surround the forespore, while pink arrows indicate detached coat layers termed “bearding.” The green arrows mark abnormal forespore shape, where the forespore appears to be pinched at the mother cell-proximal side; the yellow arrows denote areas of abnormal cortex thickness. (C) TEM analyses of sporulating JIR8094 strains. Percentage of cells with the observed phenotype based on analyses of a minimum of 50 TEM images. Download FIG S3, TIFF file, 0.6 MB. Copyright © 2017 Ribis et al. 2017 Ribis et al. This content is distributed under the terms of the Creative Commons Attribution 4.0 International license.

We also measured the impact of VM on *C. difficile* sporulating cell chloroform resistance, since a *B. subtilis VM* mutant is sensitive to chloroform (>6-log defect relative to wild type [25]). An ~4-fold reduction in chloroform resistance was observed in *C. difficile VM* mutants relative to the wild type (*P* < 0.05, Table S4 in Data Set S1), with expression of *CD25622-VM* from the *pyrE* locus again restoring chloroform resistance to wild-type levels. Taken together, *C. difficile* VM is necessary for optimal heat- and chloroform-resistant spore formation, but the requirement for VM during spore formation differs markedly between *C. difficile* and *B. subtilis*.

### Loss of VM causes morphological abnormalities in a subset of *C. difficile* sporulating cells.

Consistent with the relatively minor role that VM plays during *C. difficile* spore formation, phase-contrast microscopy revealed that *CD25622-VM*::*ermB* and *VM*::*ermB* mutants produced wild-type-like, phase-bright forespores and free spores (Fig. 2B, yellow and blue arrows, respectively). Nevertheless, these strains also produced forespores with clear morphological abnormalities. Phase-dark extensions of the phase-bright forespores were frequently observed in ~15% of sporulating *VM* mutant cells (Fig. 2B, pink arrows, and Table S5 in Data Set S1) similar to the mislocalized, polymerized coat observed in engulfment mutants lacking components of the SpoIIQ-SpoIIIA channel complex (39). *VM* mutants also produced phase-gray and phase-dark forespores with abnormal shapes (~13%) in which the forespores appeared to be pinched at the mother cell-proximal side (Fig. 2B, green arrow, and Table S5 in Data Set S1). Both these abnormal phenotypes were observed at a higher frequency in *VM* mutants than in the wild type and the complementation strains (<4%, Table S5 in Data Set S1). Given that similar morphological abnormalities were observed in *VM*::*ermB* and *CD25622-VM*::*ermB* mutants in the JIR8094 strain background (Fig. S3A), these morphological defects are likely due to loss of VM. These morphological defects would appear to differ from the phase-dark sporelets produced by *B. subtilis VM* mutants (14), but direct comparisons cannot be made because images of these sporelets have not been published.

**FIG 3:**
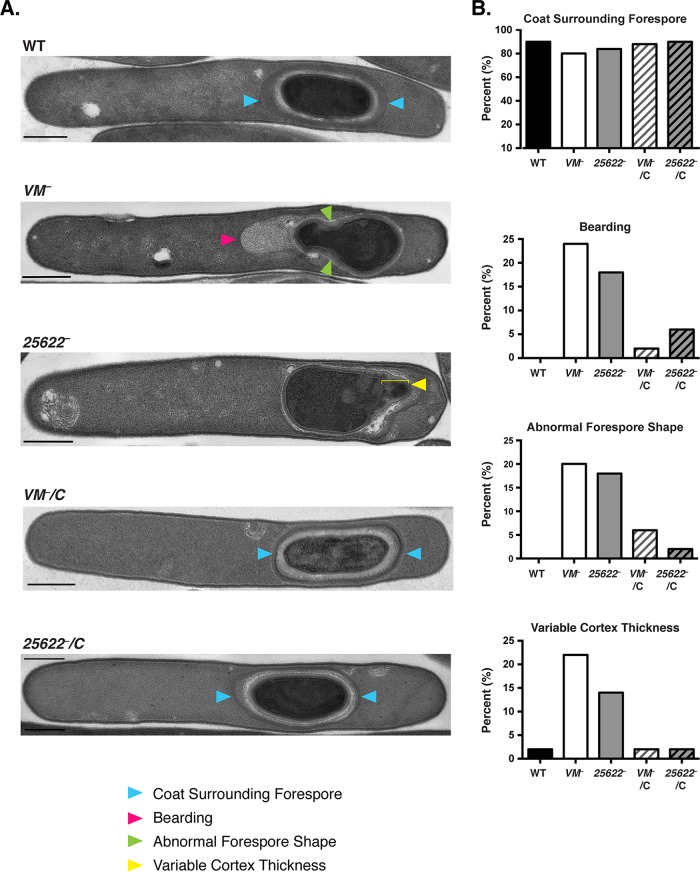
Morphological defects are visible in sporulating *VM* mutant cells. (A) Transmission electron microscopy (TEM) analyses of sporulating cultures at 23 h of wild-type (WT) 630Δ*erm*, Δ*0A*, and *VM* and *CD25622-VM* TargeTron mutants and their complements (*VM*::*ermB*/C and *25622*::*ermB*/C). Bars, 500 nm. Blue arrowheads mark coat layers that encase the forespore, while the pink arrowhead indicates detached coat layers termed “bearding.” The green arrowheads highlight abnormal forespore shape, where the forespore appears to be pinched at the mother cell-proximal side, while the yellow arrowhead denotes areas of abnormal cortex thickness. (B) Percentage of cells with the observed phenotype based on analyses of a minimum of 50 TEM images.

To assess whether polymerized coat is mislocalized in sporulating *C. difficile VM* mutant cells as predicted by the phase-contrast analyses, we used transmission electron microscopy (TEM) to visualize the multilayered coat. These analyses confirmed that polymerized coat failed to adhere to the forespore-mother cell interface in *C. difficile VM* mutants (termed “bearding” [Fig. 3A]), similar to the coat localization defects previously reported in SpoIIQ-SpoIIIA channel mutants (39). Bearding was observed in ~20% of sporulating *CD25622-VM* and *VM* mutant cells based on analyses of ≥50 images of sporulating cells that had completed engulfment; in contrast, bearding was observed in less than 6% of the complementation strains, and no bearding was observed in wild-type cells (Fig. 3B). Approximately 20% of sporulating *VM* mutant cells produced forespores that appeared to be “pinched” at one end, whereas only ~5% of forespores in the *VM* complementation strains, and none of the wild-type cells, exhibited this phenotype (Fig. 3). The frequencies of these phenotypes are slightly higher than the frequency measured using phase-contrast microscopy (Table S5 in Data Set S1), which has considerably lower resolution. TEM analyses also revealed that the *VM* mutants were more likely to produce cortex of variable thickness than the wild-type and complementation strains (Fig. 3). In total, 35% of 630Δ*erm VM* mutant cells displayed at least one of the three aberrant phenotypes (bearding, abnormal forespore shape, and variable cortex thickness). In contrast, only 2% of wild-type and 10% of the complementation strains’ sporulating cells displayed one or more of these phenotypes.

Similar morphological abnormalities were also observed in JIR8094 *VM* mutants at similar frequencies. Approximately 20% of sporulating JIR8094 *VM* mutant cells had forespores with coat bearding and/or variable cortex thickness (Fig. S3), phenotypes that were rarely observed in wild-type cells. A smaller proportion of JIR8094 *VM* mutant cells produced abnormally shaped forespores (6% of *VM*::*ermB* and 12% of *CD25622-VM*::*ermB*) relative to 630Δ*erm VM* mutant cells. However, wild-type JIR8094 also produced abnormally shaped forespores in 4% of cells (Fig. S3C), whereas wild-type 630Δ*erm* did not. Overall, 32% and 42% of sporulating JIR8094 *spoVM*::*ermB* and *CD25622-VM:ermB* cells displayed at least one of the three morphological defects compared to 11% of wild-type JIR8094 cells. Taken together, these analyses indicate that loss of VM causes some form of morphological defect during spore formation in about one-third of the population.

While cortex thickness varied within a subpopulation of sporulating *C. difficile VM* mutant cells (Fig. 3), cortex was nevertheless visible in almost all sporulating cells of this mutant. While these observations indicate that VM is dispensable for cortex production in *C. difficile*, unlike *B. subtilis* (14, 25, 27), it remains possible that *C. difficile* VM modulates cortex thickness and/or its composition.

### Loss of VM leads to a minor defect in spore purification efficiency.

**FIG 4:**
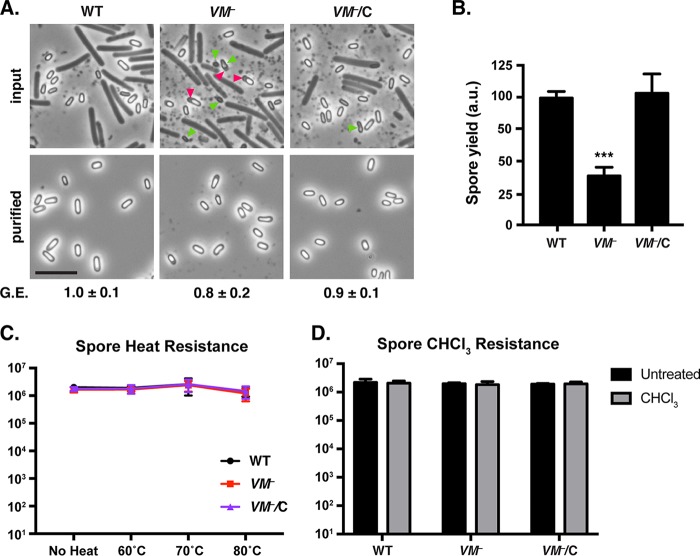
*VM* mutant spores are purified less efficiently than wild type (WT), but purified *VM* mutant spores have resistance properties similar to those of the wild type. (A) Phase-contrast microscopy images of wild-type, *VM* mutant, and *VM* mutant complementation cultures isolated 2 days after sporulation was induced (input) and spores following density gradient isolation (purified). G.E. refers to the germination efficiency of each strain relative to that of the wild type. The means and standard deviations shown are based on three biological replicates. Statistical significance relative to the wild type was determined using a one-way ANOVA and Tukey’s test. Bars, 5 µm. Examples of spores with abnormal forespore shape are marked with green arrowheads, and areas of putative mislocalized coat are marked with pink arrowheads. (B) Spore yields based on purifications from the indicated strains from four biological replicates. Yields were determined by measuring the optical density of spore purifications at 600 nm; yields are expressed in arbitrary units (a.u.). Statistical significance relative to the wild type was determined using one-way ANOVA and Tukey’s test. ***, *P* < 0.0005. (C and D) Heat (C) and chloroform (D) resistance of spores purified from the indicated strains based on three independent experiments. Numbers at left are numbers of CFUs produced by spores plated on BHIS containing taurocholate germinant.

Given that approximately one-third of *C. difficile VM* mutant cells exhibited morphological defects by phase-contrast microscopy and TEM and approximately two-thirds exhibited heat and chloroform resistance defects (Fig. 2, 3, and S3 and Table S4 in Data Set S1), we wondered whether *C. difficile VM* mutant spores would be purified less efficiently than wild-type spores. To test this possibility, we compared the spore purification yields of wild-type, *VM* mutant, and the *VM* mutant complementation strain (*VM*/C). Loss of *C. difficile* VM resulted in an ~3-fold decrease in spore yield relative to the wild type and the *VM* complementation strain (*P* < 0.001, Fig. 4A). Importantly, similar levels of sporulation were observed in the input samples, although aberrantly shaped free spores were more frequently visible in the *VM* mutant than in the wild type and the complementation strain (Fig. 4B). However, once the spores were purified on a density gradient, *VM* mutant spores were largely indistinguishable from wild-type spores by phase-contrast microscopy (Fig. 4B) and TEM analyses (Fig. S4).

10.1128/mSphere.00315-17.4 FIG S4  Transmission electron microscopy of wild-type and *VM* mutant spores. Presumed cortex layers are marked (Cx). Bars, 100 nm. Download FIG S4, TIFF file, 1 MB. Copyright © 2017 Ribis et al. 2017 Ribis et al. This content is distributed under the terms of the Creative Commons Attribution 4.0 International license.

Since the *VM* mutant exhibited an ~3-fold reduction in spore purification yield, heat resistance, and chloroform resistance relative to the wild type, we tested whether the *VM* mutant spores that survived the spore purification process would have heat and/or chloroform resistance defects. Loss of VM did not affect heat (Fig. 4C) or chloroform (Fig. 4D) resistance in purified spores, suggesting that *VM* mutant spores with morphological and functional defects fail to be purified by the density gradient.

### VM modulates, but is not essential for, IVA encasement of the *C. difficile* forespore.

**FIG 5:**
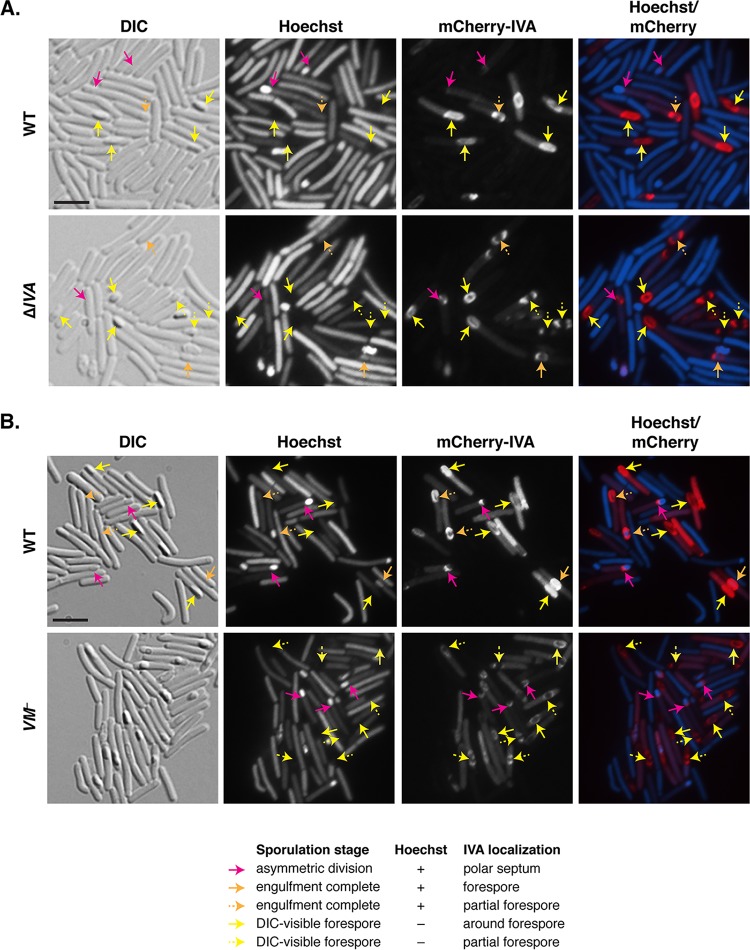
IVA can localize to and encase the forespore in the absence of VM. Fluorescence microscopy analyses of wild-type (WT) and Δ*IVA* sporulating cells (A) and wild-type and *VM* mutant sporulating cells (B) producing mCherry-IVA at 23 h post-sporulation induction. Differential interference contrast (DIC) microscopy was used to visualize forespores and free spores. The nucleoid was stained with Hoechst stain (blue), and mCherry-IVA fluorescence is shown in red. The merge of Hoechst stain and mCherry is also shown. Pink arrows highlight cells that have undergone asymmetric division but have not completed engulfment; these forespores stain with Hoechst stain (+), and mCherry-IVA localizes to the mother cell-forespore interface. Orange arrows highlight cells that appear to have completed engulfment but whose forespores retain the Hoechst dye (+). Yellow arrows mark forespores that are visible by DIC and no longer retain the Hoechst dye. Orange and yellow solid arrows indicate that mCherry-IVA fully encases the forespore, while dashed arrows indicate partial encasement of the forespore. Bars, 5 µm.

Having established that approximately two-thirds of sporulating *C. difficile VM* mutant cells have functional defects, we wondered whether the localization of VM’s putative binding partner, IVA, would be disrupted in *VM* mutant cells. To address this question, we generated a construct encoding an N-terminal fusion of mCherry to *C. difficile* IVA because a C-terminally green fluorescent protein (GFP)-tagged *B. subtilis* IVA fails to localize properly, in contrast with N-terminally GFP-tagged *B. subtilis* (21), and C-terminally His_6_-tagged *C. difficile* IVA is produced at low levels in *Escherichia coli*, in contrast with N-terminally His_6_-tagged IVA (data not shown). When mCherry-IVA was coproduced with native IVA (WT/*mCherry-IVA*), the fusion protein (i) localized to the mother cell-forespore interface of cells that had initiated engulfment (Fig. 5, pink arrows) and (ii) encased the forespore of cells that had completed engulfment (Fig. 5, yellow and orange arrows). Taken together, *C. difficile* IVA preferentially localizes to the forespore in a manner similar to *B. subtilis* IVA (21).

When the *mCherry-IVA* construct was used to complement Δ*IVA*, the mCherry-IVA signal was concentrated mostly at the forespore, although a higher proportion of the cells appeared to cap, rather than fully encase, the forespore (Fig. 5A, dashed arrows). Since these observations implied that the mCherry-IVA fusion protein was not fully functional, we measured its ability to complement the heat resistance phenotype of a Δ*IVA* strain. The resulting Δ*IVA*/*mCherry-IVA* strain produced heat-resistant spores ~5-fold less frequently than the wild type (*P* ≤ 0.05, one-way analysis of variance [ANOVA]), whereas WT/*mCherry-IVA* produced heat-resistant spores ~2-fold less frequently than the wild type (this difference was not statistically significant [Fig. S5A]). Given that mCherry-IVA was less capable of encasing the forespore in the absence of wild-type IVA and that *B. subtilis* IVA ATPase mutants exhibit similar defects in encasing the forespore (40), *C. difficile* mCherry-IVA is likely less efficient at polymerizing than wild-type IVA.

10.1128/mSphere.00315-17.5 FIG S5  Complementation of Δ*IVA* by mCherry-IVA and N491G IVA. (A) Phase-contrast microscopy of wild-type 630Δ*erm* and Δ*IVA* strains complemented with either IVA, mCherryIVA, or N491G IVA, and Δ*0A* after 22 h of sporulation. Examples of phase-bright forespores and spores are marked using yellow and blue arrows, respectively. Mislocalized coat regions are marked by pink arrows. HR refers to the heat resistance of each strain relative to the wild type. The means and standard deviations shown are based on three biological replicates. Statistical significance relative to the wild type was determined using one-way ANOVA and Tukey’s test. Bars, 5 µm. (B) Western blot analyses of the strains in panel A using anti-IVA and anti-mCherry antibodies. Nonspecific bands are marked with asterisks. A small amount of mCherry-IVA undergoes cleavage during sporulation, appearing to liberate IVA and mCherry. Download FIG S5, TIFF file, 0.6 MB. Copyright © 2017 Ribis et al. 2017 Ribis et al. This content is distributed under the terms of the Creative Commons Attribution 4.0 International license.

Although most of the mCherry-IVA signal localized to the forespores of WT/*mCherry-IVA* and Δ*IVA*/*mCherry-IVA*, cytosolic mCherry signal was also observed (Fig. 5A). This cytosolic signal likely results from the small amount of mCherry that is liberated upon processing of mCherry-IVA (Fig. S5B). Taken together, these results confirm that *C. difficile* IVA encases the forespore and that this localization pattern correlates with functional spore formation.

With the functional mCherry-IVA reagent in hand, we next assessed the impact of VM on the localization of mCherry-IVA. The fusion protein localized primarily around the forespore in the absence of VM (Fig. 5B), although 29% of *VM* mutant cells exhibited partial encasement by mCherry-IVA around the forespore. In contrast, 14% of wild-type cells exhibited this phenotype based on analyses of 100 sporulating cells that had completed engulfment. These results strongly suggest that *C. difficile* IVA can still localize to and surround the forespore in the absence of *C. difficile* VM, albeit with somewhat lower efficiency than the wild type. They also contrast with the localization pattern of *B. subtilis* IVA, which forms foci on the forespore but fails to encase it in ~30% of *VM* mutant cells (22, 23), relative to ~5% of wild-type cells. Unfortunately, attempts to localize VM during sporulation using fluorescent protein fusions have thus far been unsuccessful.

### *C. difficile* VM binds IVA *in vitro*.

Since these data indicated that IVA can localize to and surround the forespore even in the absence of VM in the majority of sporulating cells, we wondered whether *C. difficile* VM even binds *C. difficile* IVA. In *B. subtilis*, VM binding to IVA depends on Ile6 in VM and Gly486 in IVA, as a G486V mutation in IVA can rescue the sporulation defect of the I6A VM mutant (22). Interestingly, while Ile6 is conserved in *C. difficile* VM (Fig. 1), Gly486 is an asparagine in *C. difficile* IVA (Asn491), other *Peptostreptococcaceae* spp. (41), and *Clostridium perfringens* IVA (Fig. S6).

10.1128/mSphere.00315-17.6 FIG S6  Sequence alignment of the C-terminal domain (68) of IVA homologs in the *Bacilli* and *Clostridia*. Residues that are completely conserved are boxed in blue with white text. Conserved identical residues are boxed in green, and conserved similar residues are boxed in yellow. The residues corresponding to Gly486 in *B. subtilis* IVA are outlined in red. Mutation of Gly486 to valine suppressed the sporulation defect of an I6A VM mutant and restored IVA’s ability to encase the forespore in the I6A VM mutant (22). SpoIVA sequence accession numbers: *B. subtilis*, NP_390161; *Bacillus licheniformis*, AAU23942; *Paenibacillus* sp., GYMC10_2192; *Bacillus clausii*, WP_011246725; *Clostridium acetobutylicum*, NP_348339; *Clostridium perfringens*, ABG84677; *Clostridium beijerinckii*, WP_026889165; *Clostridium butyricum*, EDT74816; *Clostridium novyi*, KEH85260; *Clostridium sporogenes*, EHN14425; *Clostridium bartlettii*, CDA10929; *Clostridium bifermentans*, WP_021433867; *Clostridium mangenotii*, WP_024621862; *Peptostreptococcaceae* sp., WP_026900372; *Clostridium sordellii*, CEQ01088; *C. difficile*, CAJ69515. Download FIG S6, TIFF file, 0.4 MB. Copyright © 2017 Ribis et al. 2017 Ribis et al. This content is distributed under the terms of the Creative Commons Attribution 4.0 International license.

**FIG 6:**
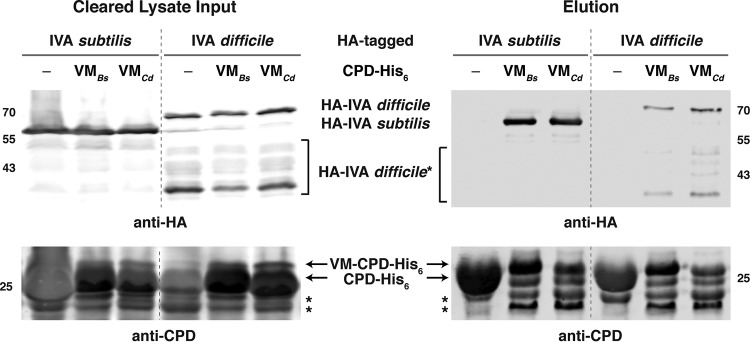
*C. difficile* VM directly binds IVA in coaffinity purification analyses. HA-tagged *B. subtilis* and *C. difficile* IVA were coproduced in *E. coli* with either VM-CPD-His_6_ fusions, where VM was from either *B. subtilis* (*Bs*) or *C. difficile* (*Cd*), or CPD-His_6_ alone. Cleared (soluble) lysate samples were prepared from the cultures following lysis by sonication and centrifugation. CPD-His_6_ variants were affinity purified, and high imidazole was used to elute the His-tagged proteins and any interacting partners. Cleared lysate and elution samples were analyzed by Western blotting using anti-HA and anti-CPD (42) antibodies. Degradation products of HA-tagged *C. difficile* IVA are highlighted by the square brackets, while degradation products of VM-CPD-His_6_ are highlighted by asterisks. Numbers at left and right of the gels indicate molecular masses in kilodaltons.

To test whether *C. difficile* VM can bind IVA, we coproduced His_6_-tagged *C. difficile* VM (VM_*Cd*_) with hemagglutinin (HA)-tagged *C. difficile* IVA in *E. coli* and measured the ability of HA-tagged IVA to copurify with His_6_-tagged VM_*Cd*_ using Ni^2+^-affinity chromatography. To facilitate the production and detection of VM_*Cd*_, we fused it to CPD-His_6_, an inducible, self-cleaving protease domain (42) that can promote the production and purification of small amphipathic helices (43, 44). As a positive control, we measured the ability of HA-tagged *B. subtilis* IVA to copurify with *B. subtilis* VM-CPD-His_6_. As negative controls, we tested whether the HA-tagged IVA variants could copurify with CPD-His_6_. Last, we also tested whether HA-tagged *C. difficile* IVA could bind *B. subtilis* VM-CPD-His_6_ and vice versa. When VM_*Cd*_-CPD-His_6_ was pulled down on nickel-nitrilotriacetic acid (Ni-NTA) beads in the presence of either *B. subtilis* or *C. difficile* HA-tagged IVA, both HA-tagged IVA variants were detected in the imidazole elution fraction (Fig. 6). Since VM_*Bs*_-CPD-His_6_ also pulled down both HA-tagged IVA variants, whereas CPD-His_6_ failed to pull down HA-tagged *B. subtilis* and *C. difficile* IVA from the cleared lysates, these results indicate that *C. difficile* IVA can interact specifically with both *B. subtilis* and *C. difficile* VM in this heterologous system.

Despite these observations, both VM-CPD-His_6_ fusions pulled down more *B. subtilis* IVA than *C. difficile* IVA, so we wondered whether the bulkier Asn491 residue in *C. difficile* IVA diminished its ability to bind VM relative to *B. subtilis* IVA, which carries a glycine (G486) at this position. To test this possibility, we coproduced HA-tagged *C. difficile* N491G IVA with VM-CPD-His_6_. Unfortunately, N491G IVA was degraded more readily than wild-type *C. difficile* IVA in *E. coli* (data not shown), so we could not assess whether the N491G mutation enhanced binding to VM. However, when we produced N491G IVA in the Δ*IVA* background, N491G IVA was detected at wild-type levels and no change in the heat resistance of Δ*IVA*/N491G *IVA* was observed (Fig. S5). The latter observation appears to be consistent with the finding that a G486V IVA mutation in *B. subtilis* does not alter heat-resistant spore formation (22). Taken together, our results indicate that, despite *C. difficile* VM’s ability to bind IVA, disrupting this interaction in *C. difficile* has a relatively minor impact on functional spore formation.

## DISCUSSION

In *B. subtilis*, the proper assembly of the coat basement layer is essential for both coat and cortex assembly and thus functional spore formation (8, 11). While extensive analyses have identified key regulators of this process in *B. subtilis*, namely, VM, IVA, CmpA, and VID (11, 33, 45), major differences in basement layer coat morphogenesis exist between *C. difficile* and *B. subtilis*. These include the lack of CmpA and VID homologs in the *Clostridia* (26, 33), the lack of SipL homologs in the *Bacilli* (34), the dispensability of IVA for cortex formation in *C. difficile* (34), and the ability of *C. difficile* IVA but not *B. subtilis* IVA to bind LysM domains (23, 34). In the present study, we identified another major difference in spore assembly between *C. difficile* and *B. subtilis*: VM is essential for *B. subtilis*, but not *C. difficile*, spore formation. Whereas *B. subtilis* VM mutants exhibit ~6-log reductions in spore purification yields (26) and heat and chloroform resistance (25), only ~3-fold decreases in these properties were observed in *C. difficile* (Fig. 2 and 3; see also Table S4 in Data Set S1). Thus, this study contributes to a growing body of work indicating that *C. difficile* and *B. subtilis* can have differential requirements for conserved factors during sporulation (39, 46, 47).

Our finding that loss of VM does not abolish functional spore formation in *C. difficile* was somewhat surprising, since (i) key functional residues of *B. subtilis* VM are conserved in *C. difficile* (Fig. 1); (ii) *C. difficile* VM and *C. difficile* IVA directly interact (Fig. 6), similarly to *B. subtilis* VM and IVA (22); and (iii) *VM* is transcribed in the mother cell early during sporulation in both *B. subtilis* (37) and *C. difficile* (Fig. 2 and S1). A potential reason why VM may be differentially required between these two organisms is that *B. subtilis* IVA, but not *C. difficile* IVA, depends on VM for forespore encasement (Fig. 5B). In *B. subtilis*, sporulating cells that fail to encase IVA around the forespore (due to loss of VM [23] or IVA ATPase activity [40]) are lysed by a CmpA-dependent checkpoint mechanism (26). While IVA fails to surround the forespore in almost all *B. subtilis VM* mutant cells, only ~30% of *C. difficile VM* mutant cells exhibit IVA encasement defects (Fig. 5B). Thus, even if *C. difficile* employed a checkpoint mechanism analogous to the one activated by CmpA, it would not be strongly induced in a *C. difficile VM* mutant.

Clearly, a major difference between *B. subtilis* and *C. difficile* IVA is their dependence on VM for IVA forespore encasement. *C. difficile* IVA may be able to independently interact with the forespore membrane largely independently of VM, such that VM is largely dispensable in *C. difficile*. Alternatively, a redundant factor could help IVA surround the forespore in the absence of VM. While additional possibilities exist, we posit that *C. difficile* has more redundant factors and fewer checkpoint mechanisms than *B. subtilis* to maximize spore production prior to exiting the host (33, 48). Whereas spore formation is essential for *C. difficile* to survive exit from the host (5), this process is only one of several alternative lifestyles that can be adopted by *B. subtilis* (49). As a result, mechanisms may have evolved to ensure the fidelity of this developmental process (33, 48).

While addressing these questions awaits further analysis, another question raised by this study is why ~70% of *VM* mutant spores are sensitive to heat and chloroform (Fig. 2; Table S4 in Data Set S1) when only ~30% of *VM* mutant cells exhibit gross morphological defects (Fig. 3 and 5). Since the cortex is critical for spores to resist heat and chloroform (9, 10), heat- and chloroform-sensitive *C. difficile VM* mutant cells may have cortex defects that are not visible by TEM. Indeed, we found that ~20% of *VM* mutant cells produce cortex with various thicknesses (Fig. 3 and S3), suggesting that *C. difficile* VM alters the activities of the cortex synthesis and/or modification machinery. However, little is known about the factors controlling cortex production and/or modification in *C. difficile*, although homologs of the *B. subtilis* cortex synthesis machinery (SpoVB, SpoVD, and SpoVE [50]) and cortex-modifying enzymes (CwlD and PdaA [51, 52]) are conserved in *C. difficile*. Future studies of cortex production and modification in *C. difficile* could identify mechanisms by which VM modulates spore formation and may also provide insight into the mechanisms underlying the forespore pinching observed in ~20% of *VM* mutant cells (Fig. 3).

Whether such mechanisms are related to how *B. subtilis* links basement layer formation to cortex assembly (27, 33), which is also poorly defined, awaits further investigation. Regardless, our study further highlights that the coat assembly pathway in *C. difficile* exhibits substantial differences relative to *B. subtilis* and raises questions as to how these functional differences evolved for the shared coat morphogenetic proteins IVA and VM. Analyses of coat assembly in other clostridial organisms may provide insight into this question.

## MATERIALS AND METHODS

### Bacterial strains and growth conditions.

JIR8094 (630E [53]) and 630Δ*erm*Δ*pyrE* (38) were used as the parental strains for TargeTron-based gene disruption (54). 630Δ*erm*Δ*pyrE* was also used for *pyrE-*based allele-coupled exchange (ACE [38]). *C. difficile* strains are listed in Table S1 in Data Set S1 and were grown on BHIS agar (55) supplemented with taurocholate (TA; 0.1% [wt/vol]; 1.9 mM), kanamycin (50 µg/ml), cefoxitin (8 µg/ml), FeSO_4_ (50 µM), and/or erythromycin (10 µg/ml) as needed. *C. difficile* defined minimal medium (CDMM [56]) was supplemented with 5-fluoroorotic acid (5-FOA) at 2 mg/ml and uracil at 5 µg/ml as needed for ACE. Cultures were grown under anaerobic conditions using a gas mixture containing 85% N_2_, 5% CO_2_, and 10% H_2_.

*Escherichia coli* strains for HB101/pRK24-based conjugations and BL21(DE3)-based protein production are listed in Table S1 in Data Set S1. *E. coli* strains were grown at 37°C with shaking at 225 rpm in Luria-Bertani broth (LB). The medium was supplemented with chloramphenicol (20 µg/ml), ampicillin (50 µg/ml), or kanamycin (30 µg/ml) as indicated.

### *E. coli* strain construction.

All primers are listed in Table S2 in Data Set S1. For constructing targeting sequences for *spoVM* and *CD25622-VM*, a modified plasmid containing the retargeting group II intron, pCE245 (a gift from C. Ellermeier, University of Iowa), was used as a template. For targeting *spoVM*, primers 1094, 1095, 1096, and 532 (EBS universal primer; Sigma-Aldrich) were used; for targeting *CD25622*, primers 2186, 2187, 2188, and 532 were used. The resulting sequences were digested with BsrGI and HindIII and cloned into pJS107 (6), a derivative of pJIR750ai (Sigma-Aldrich). Ligations were transformed into DH5α, and the resulting plasmids were transformed into HB101/pRK24.

To construct the *CD25622-VM* complementation plasmid for ACE, primer pair 2148 and 2149 was used to amplify a region 77 bp upstream of *CD25622* and 9 bp downstream of *spoVM*. To construct the *spoIVA* complementation constructs, primer pair 2036 and 2037 was used to amplify 80 bp upstream and 159 bp downstream of *spoIVA*. To construct the N491G *spoIVA* complementation construct, splicing-by-overlap-extension (SOE) primer pair 2177 and 2178 was used in conjunction with 2036 and 2037. To construct the *mCherry-spoIVA* construct, primer pair 2202 and 2203 was used to amplify the 80-bp upstream region of *spoIVA*, while primer pair 2204 and 2205 was used to amplify a codon-optimized mCherry construct (a kind gift of D. Weiss and C. Ellermeier [57]). The resulting PCR products fuse the *spoIVA* promoter region to codon-optimized *mCherry* (57) and the *spoIVA* gene when assembled into pMTL-YN1C digested with NotI and XhoI using Gibson assembly (58).

To construct the pMTL-YN3 Δ*spoIVA* allelic exchange construct, primer pair 2272 and 2273 was used to amplify regions ~1 kb upstream and downstream of *spoIVA*. This primer pair was used in conjunction with the SOE primer pair 1746 and 1747, which fuses the first 12 codons of *spoIVA* to its last 13 codons. The resulting PCR products were ligated into AscI-SbfI-digested pMTL-YN3 using Gibson assembly.

To create the recombinant protein expression constructs for the coaffinity purifications, primer pair 1086 and 1609 was used to amplify *spoVM* lacking the stop codon using *C. difficile* genomic DNA as the template. The resulting PCR product was digested with NdeI/SalI and ligated to pET22b-Δ50-CPD (43) to construct a SpoVM-CPD-His_6_ fusion construct. The *B. subtilis* SpoVM-CPD expression construct was cloned in a similar manner except that the *spoVM* gene was digested from pUC57-Kan-*spoVM subtilis* (synthesized by GenScript) using NdeI/SalI. To construct the HA-tagged *B. subtilis spoIVA* expression construct, primer pair 1318 and 1237 was used to amplify *spoIVA* encoding an N-terminal HA tag using *B. subtilis* DNA as a template. The resulting PCR product was digested with NheI and XhoI and ligated into pET28a digested with the same enzymes. DNA sequencing was used to confirm all the constructs described.

### *C. difficile* strain construction.

TargeTron-based gene disruption using pJS107-*spoVM* and pJS107-*CD25622* inserted into either JIR8094 or 630Δ*erm*Δ*pyrE* was employed as previously described (54, 59). Primer pair 2148 and 2149 was used to screen isolated erythromycin-resistant colonies for TargeTron (2-kb) insertions in *spoVM* and *CD25622*. Allele-coupled exchange (ACE [38]) was used as previously described (46) to construct the clean *spoIVA* deletion in 630Δ*erm*Δ*pyrE*. Primer pair 1743 and 1744 was used to screen isolated FOA-resistant, uracil auxotroph colonies for the Δ*spoIVA* deletion. At least two clones of each mutant strain were phenotypically characterized.

### *C. difficile* complementation.

The *pyrE* locus was restored using pMTL-YN1C and pMTL-YN1C-based complementation constructs as previously described (46). At least two independent clones from each complementation strain were phenotypically characterized.

### Sporulation.

*C. difficile* strains were grown from glycerol stocks on BHIS plates containing TA (0.1% [wt/vol], 1.9 mM). Colonies from these plates were used to inoculate liquid BHIS cultures, which were grown to stationary phase either for several hours or overnight. The liquid cultures were back-diluted 1:50 into BHIS, and cultures were grown until they reached an optical density at 600 nm (OD_600_) between 0.35 and 0.7. One hundred to 150 µl of this culture was used to inoculate 70:30 agar plates (40 ml [34]), and sporulation was induced on this medium for 20 to 24 h. The constant thickness of the 70:30 plates combined with the broth culture inoculation reduced the variability in heat resistance efficiencies between replicates.

### Phase-contrast microscopy.

Sporulating cells were enumerated for apparent mislocalized coat and misshapen forespores using phase-contrast microscopy. A minimum of 400 cells (includes sporulating cells and free spores) from at least two independent replicates was counted, and the percentages of phase-bright forespores, phase-dark forespores, phase-dark extensions, and misshapen forespores were determined relative to the total number of sporulating cells. Free spores were also enumerated.

### qRT-PCR.

Transcript levels for *spoVM* and *rpoB* were determined using cDNA templates prepared from five biological replicates of wild-type JIR8094, *spo0A* mutant, *sigE* mutant, *spoIIID* mutant, and *sigK* mutant strains (36, 59). Primer pair 1086 and 1087 was used to amplify the *spoVM* coding sequence, while the housekeeping gene *rpoB*-specific primers have been previously described (36). qRT-PCR was performed as previously described (36) using SYBR green to quantify transcript levels for both *spoVM* and *rpoB*. Transcript levels were normalized to *rpoB* using the standard curve method and calculated relative to *spo0A* mutant.

### Heat resistance assay on sporulating cells.

*C. difficile* strains were induced to sporulate for 20 to 24 h as described above. Heat-resistant spore formation was measured as previously described (60). Heat resistance efficiencies represent the average ratio of heat-resistant cells for a given strain to the average ratio determined for the wild type based on a minimum of three biological replicates.

### Chloroform resistance assay.

Following the induction of sporulation for 20 to 24 h, a quarter of the 70:30 plate was harvested into phosphate-buffered saline (PBS). Sporulating cells were either mock treated or exposed to 10% chloroform for 15 min with periodic mixing, after which the sample was serially diluted and plated on BHIS-TA plates. Chloroform resistance was calculated by determining the ratio of chloroform-resistant cells to untreated cells and averaging this ratio across four biological replicates. Chloroform resistance efficiencies were determined by comparing the average chloroform resistance for a given strain to that of the wild type.

### TEM analyses.

Sporulating cultures (24 h) were fixed and processed for electron microscopy by the University of Vermont Microscopy Center as previously described (34). A minimum of 50 full-length sporulating cells were used for phenotype counting.

### Spore purification.

Sporulation was induced on four 70:30 plates as described above for 2 to 3 days. Sporulating cells were scraped into ice-cold water, and a sample was removed to analyze the spore input by phase-contrast microscopy. Spores were purified as previously described (61). Ice-cold water washes were used to lyse sporulating cells, after which released DNA was removed by DNase, and spores were purified on a HistoDenz density gradient and resuspended in equal volumes of water. Purified spores were analyzed by phase-contrast microscopy to ensure that they were >95% pure, and spore yields were determined by measuring the optical density (600 nm) of the spore purifications. Spores were stored in water at 4°C.

### Germination assay.

Purified spores (~1 × 10^7^ spores, equivalent to 0.35 OD_600_ units) were resuspended in 100 µl of water, and 10 µl was removed for serial dilutions and plating on BHIS-TA. Colonies arising from germinated spores were counted between 20 and 24 h. Germination efficiency represents the average number of CFU produced by spores of a given strain on BHIS-TA relative to the average number produced by wild-type spores based on three biological replicates of at least two independent spore preparations.

### Spore heat titration.

Approximately 4 × 10^7^ spores (1.5 OD_600_ units) were resuspended in 420 µl of water. One-hundred-microliter aliquots were removed for the untreated sample and three temperature treatments. Spores were incubated at either 60°C, 70°C, or 80°C for 15 min, and 10-µl aliquots were removed for serial dilution and plating onto BHIS-TA plates similarly to previous studies (46).

### Spore chloroform resistance.

Approximately 2 × 10^7^ spores (0.75 OD_600_ units) were resuspended in 190 µl water. Ninety microliters was then added to tubes containing either 10 µl of water or chloroform. Spores were incubated for 15 min, after which 10 µl of the sample was serially diluted and plated on BHIS-TA plates similarly to previous studies (62).

### Fluorescence microscopy.

Live cell fluorescence microscopy was performed using Hoechst 33342 (Molecular Probes; 15 µg/ml) and mCherry protein fusions to localize IVA. Samples were prepared on agarose pads as previously described (39) except that the samples were not imaged until 30 min after harvesting, since this time frame allowed for reconstitution of mCherry fluorescence signal in the anaerobically growing bacteria. Sporulating cells were exposed to ambient oxygen for a maximum of 80 min to minimize DNA fragmentation; no cell lysis was observed under these conditions. Differential interference contrast (DIC) and fluorescence microscopy were performed using a Nikon PlanApo Vc 100× oil immersion objective (1.4 numerical aperture [NA]) on a Nikon Eclipse Ti2000 epifluorescence microscope. An EXi Blue Mono camera (QImaging) with a hardware gain setting of 2.0 was used to acquire multiple fields for each sample in 14-bit format with 2-by-2 binning using NIS-Elements software (Nikon). The Texas Red channel was used to acquire images after a 300- to 400-ms exposure, 75- to 90-ms exposures were used to visualize the Hoechst stain, and ~10- to 20-ms exposures were used for DIC microscopy. Twenty-megahertz images were subsequently imported into Adobe Photoshop CS6 for minimal adjustments in brightness/contrast levels and pseudocoloring.

### Western blot analyses.

Samples for immunoblotting were prepared as previously described (61). Briefly, pelleted sporulating cells were resuspended in 100 µl of PBS, and 50-µl samples were freeze-thawed for three cycles and then resuspended in 100 µl EBB buffer (8 M urea, 2 M thiourea, 4% [wt/vol] SDS, 2% [vol/vol] β-mercaptoethanol). The samples were boiled for 20 min, pelleted at high speed, resuspended in the same buffer to maximize protein solubilization, boiled for another 5 min, and then pelleted at maximum speed. Samples were resolved on 12% SDS-PAGE gels, transferred to an Immobilon-FL polyvinylidene difluoride (PVDF) membrane, and blocked in Odyssey Blocking buffer with 0.1% (vol/vol) Tween 20. Mouse anti-SpoIVA (63) and rabbit anti-mCherry (Abcam, Inc.) were used at 1:2,500 and 1:2,000 dilutions. IRDye 680CW and 800CW infrared dye-conjugated secondary antibodies were used at a 1:20,000 dilution, and blots were imaged on an Odyssey LiCor CLx imaging system.

### Coaffinity purification assays.

VM-CPD-His_6_ variants and associated IVA variants were purified on Ni^2+^-affinity resin from 500 ml of 2YT (0.55% NaCl, 1.0% yeast extract, 1.5% [wt/vol] tryptone) culture as previously described (64). Input samples were taken from cultures following isopropyl-β-d-thiogalactopyranoside (IPTG) induction at 18°C for 15 h. Culture pellets were resuspended in ~25 ml lysis buffer (500 mM NaCl, 50 mM Tris-HCl, pH 7.5, 15 mM imidazole, 10% [vol/vol] glycerol), flash frozen in liquid nitrogen, thawed, sonicated to lyse the sample, and centrifuged to clear insoluble material. Ni-NTA agarose beads (0.5 ml; 5 Prime) were added to the cleared lysate for 3 h. The resin was washed extensively in lysis buffer, and His_6_-tagged proteins were eluted in 175 µl of high-imidazole buffer (500 mM NaCl, 50 mM Tris, pH 7.5, 175 mM imidazole, 10% glycerol) after nutating the sample for 5 to 10 min.
