# Supplementary figures and images for: The Conserved Spore Coat Protein SpoVM Is Largely Dispensable in Clostridium difficile Spore Formation
Source: mSphere. 2017 Sep 20;2(5):e00315-17. doi: 10.1128/mSphere.00315-17 (PMC5607322; doi:10.1128/mSphere.00315-17)

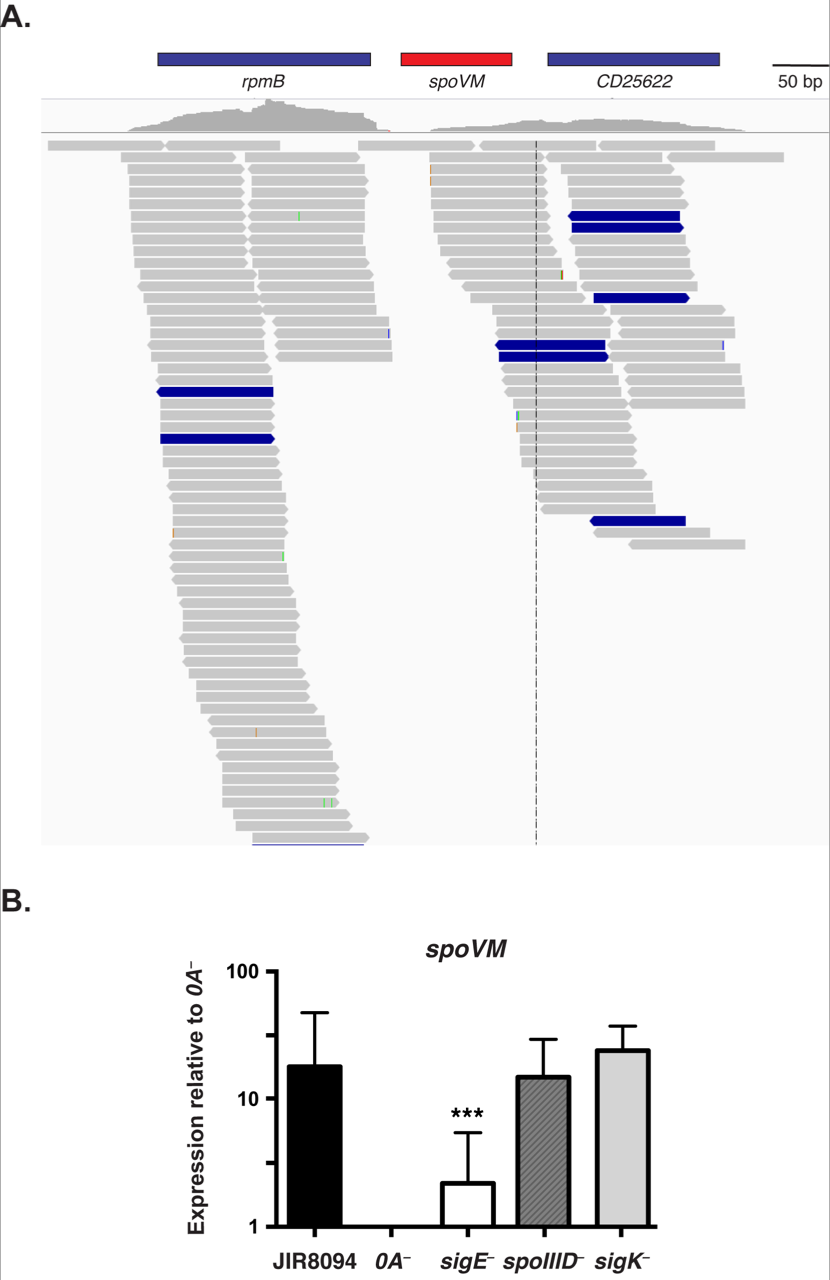

Supplement: FIG S1 [file sph005172364sf1.tiff]

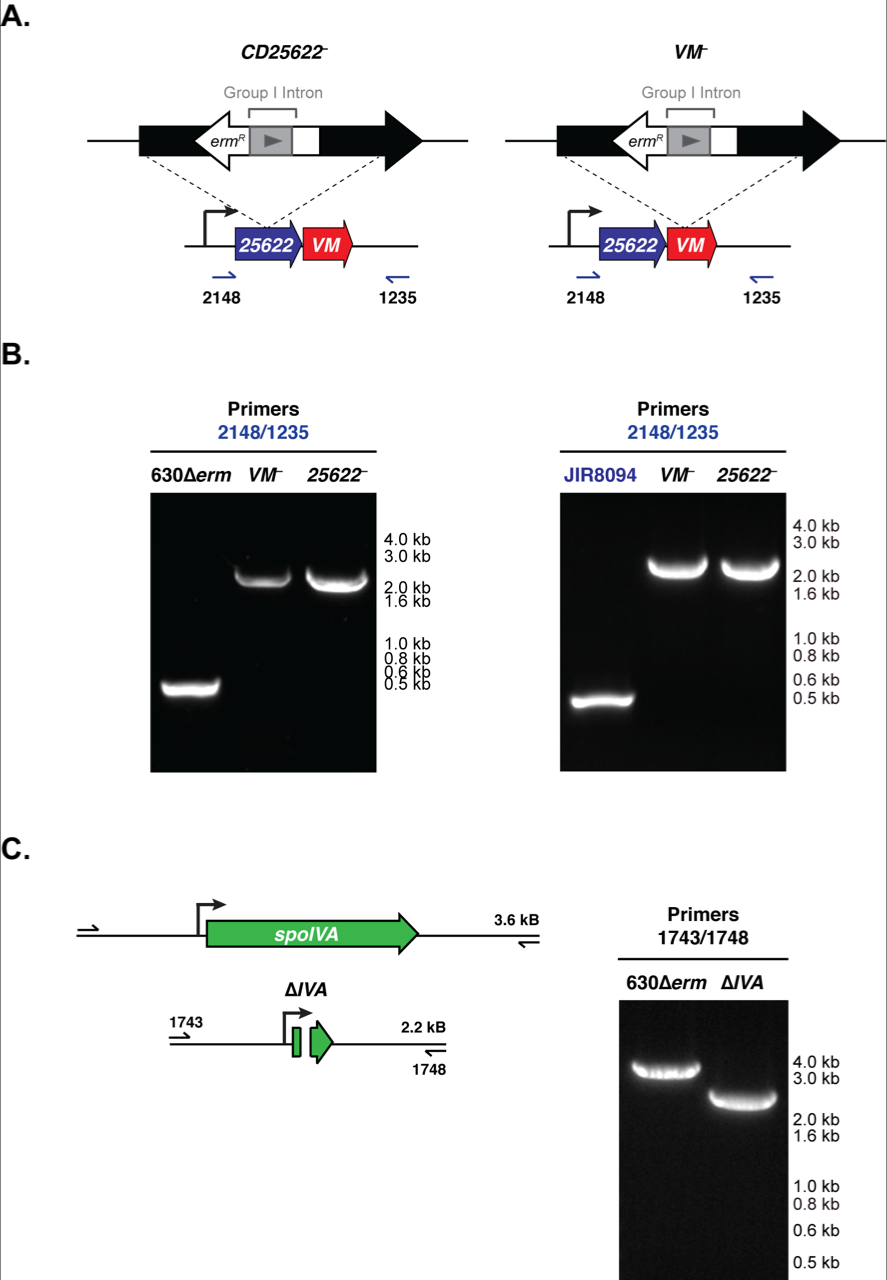

Supplement: FIG S2 [file sph005172364sf2.tiff]

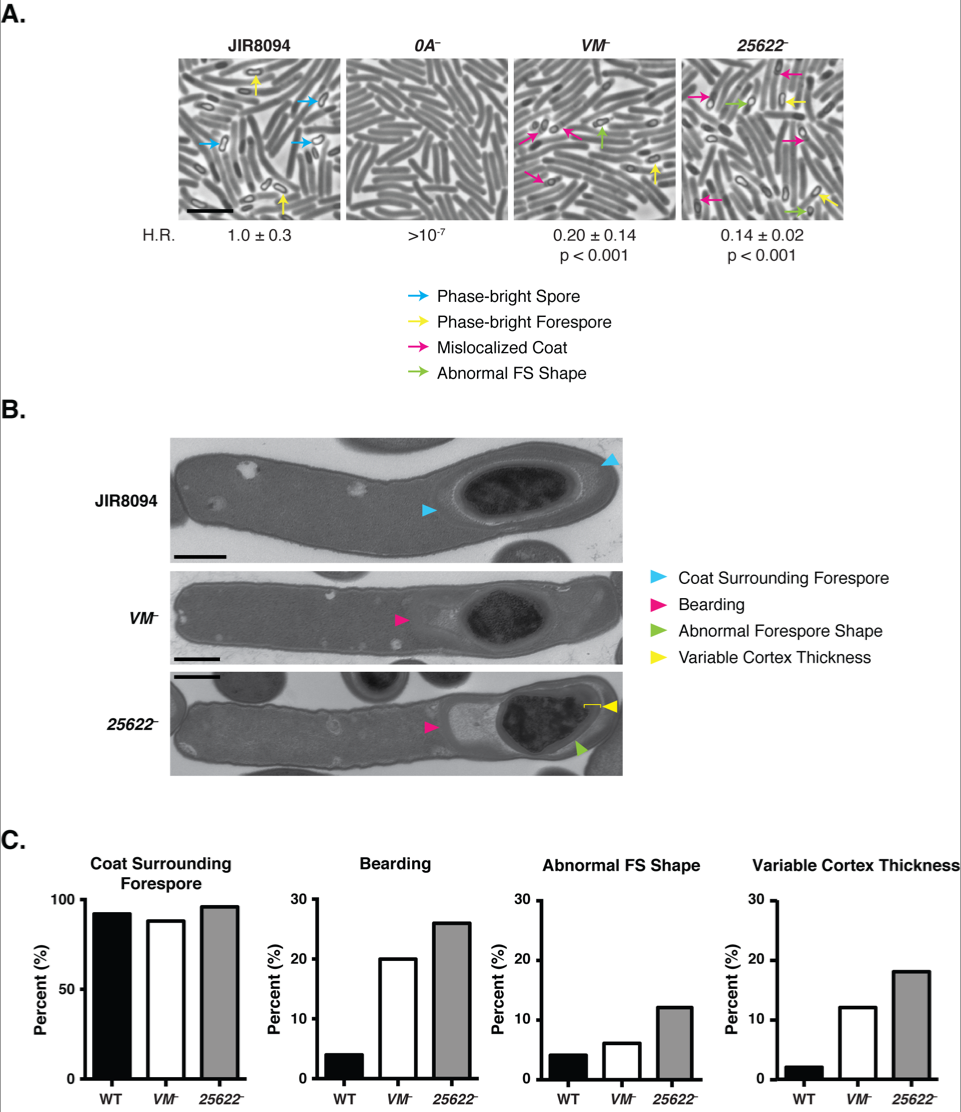

Supplement: FIG S3 [file sph005172364sf3.tiff]

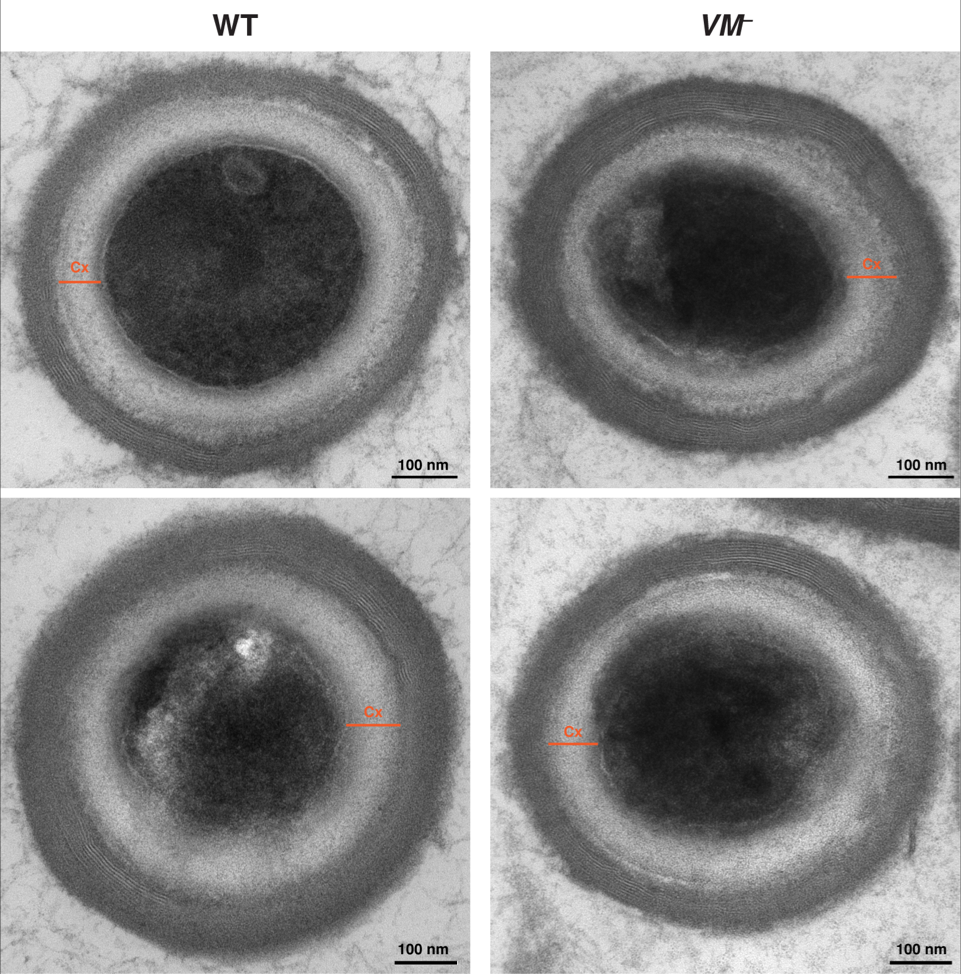

Supplement: FIG S4 [file sph005172364sf4.tiff]

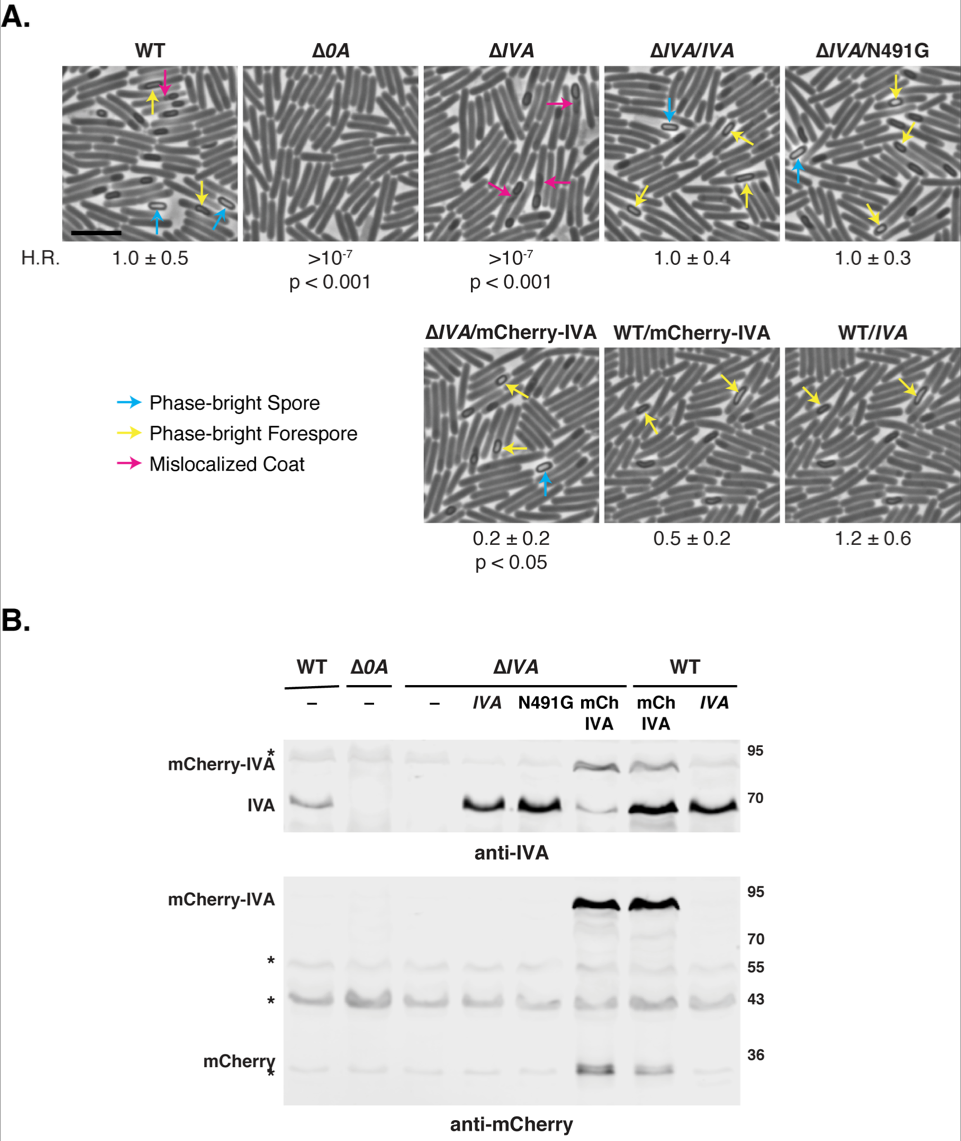

Supplement: FIG S5 [file sph005172364sf5.tiff]

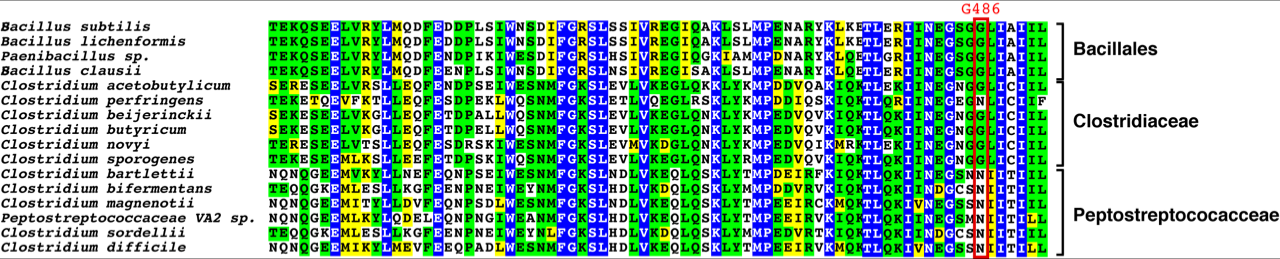

Supplement: FIG S6 [file sph005172364sf6.tiff]
