# Supplementary material for: The Conserved Spore Coat Protein SpoVM Is Largely Dispensable in Clostridium difficile Spore Formation
Source: mSphere. 2017 Sep 20;2(5):e00315-17. doi: 10.1128/mSphere.00315-17 (PMC5607322; doi:10.1128/mSphere.00315-17)
Supplement: DATA SET S1 [file sph005172364sd7.pdf]

Table S1. Strains and plasmids used in this study

**Strain #**	**Strain name**	**Relevant genotype or features**	**Source/reference**
***C. difficile* strains – JIR8094**			
11	JIR8094	*erm*-sensitive derivate of 630	(1)
282	JIR8094 *spoVM*$^{-}$	JIR8094 *spoVM*::*ermB*	This study
1135	JIR8094 *CD25622*$^{-}$	JIR8094 *CD25622*::*ermB*	This study
C. difficile* strains – 630Δ*erm			
756	630Δ*erm*Δ*pyrE*	*erm*-sensitive derivate of 630 with a deletion in *pyrE*	(2)
803	630Δ*erm*Δ*pyrE*Δ*IVA*	630Δ*erm*Δ*pyrE* with *spoIVA* deleted	This study
846	630Δ*erm*-p	*erm*-sensitive derivate of 630 with *pyrE* restored	(3)
880	630Δ*erm*Δ*IVA*-p	630Δ*erm*Δ*spoIVA* with *pyrE* restored	This study
883	630Δ*erm*Δ*IVA*/*IVA*	630Δ*erm*Δ*spoIVA* with *spoIVA* in the *pyrE* locus	This study
974	630Δ*erm*/*IVA*	630Δ*erm* with *spoIVA* in the *pyrE* locus	This study
1123	630Δ*erm*Δ*IVA*/N491G *IVA*	630Δ*erm*Δ*spoIVA* with N491G *spoIVA* in the *pyrE* locus	This study
1144	630Δ*erm*/*mCherry-IVA*	630Δ*erm* with *mCherry-IVA* in the *pyrE* locus	This study
1147	630Δ*erm*Δ*IVA*/*mCherry-IVA*	630Δ*erm*Δ*spoIVA* with *mCherry-spoIVA* in the *pyrE* locus	This study
1220	630Δ*erm*Δ*pyrE VM*$^{-}$	630Δ*erm*Δ*pyrE spoVM::ermB*	This study
1222	630Δ*erm*Δ*pyrE 25622*$^{-}$	630Δ*erm*Δ*pyrE CD25622::ermB*	This study
1269	630Δ*erm 25622*$^{-}$/C	630Δ*erm CD25622::ermB* with *CD25622-spoVM* in the *pyrE* locus	This study
1275	630Δ*erm VM*$^{-}$/C	630Δ*erm spoVM::ermB* with *CD25622-spoVM* in the *pyrE* locus	This study
1284	630Δ*erm 25622*$^{-}$-p	630Δ*erm CD25622::ermB* with *pyrE* locus restored	This study
1286	630Δ*erm VM*$^{-}$-p	630Δ*erm spoVM::ermB* with *pyrE* locus restored	This study
***E. coli* strains**			
41	DH5α	F– Φ80*lac*ZΔM15 Δ(*lacZYA-argF*) U169 *recA1 endA1 hsdR17* (rK^{-}, mK^{+}) *phoA supE44* λ– *thi-1 gyrA96 relA1*	D. Cameron
195	pET22b-Δ50CPD	pET22b-Δ50CPD in DH5 α	(4)
531	HB101/pRK24	F- *mcrB mrr hsdS20*(rB^{-}mB^{-}) *recA13 leuB6 ara-13 proA2 lavYI galK2 xyl-6 mtl-1 rpsL20* carrying pRK24	C. Ellermeier
759	pET29a/HA-*spoIVA*	pET29a with HA-*spoIVA* from *C. difficile* in DH5α	(5)
957	pJS107/*spoVM*	pJS107-*spoVM* targeting 32 bp in HB101/pRK24	This study
1441	pET22b *spoVM-CPD*	pET22bΔ50-CPD with *spoVM C. difficile* in BL21(DE3)	This study
1473	pET28a-HA-*spoIVA B.s.*	pET28a with HA-*spoIVA* from *B. subtilis* in HB101/pRK24	This study
1481	pUC57Kan-*spoVM-B.s.*	pUC57Kan with *spoVM B.subtilis-CPD*	This study
1485	pET22b *spoVM B. subt-CPD*	pET22bΔ50-CPD with *spoVM B. subtilis* in BL21(DE3)	This study
1539	pMTL-YN3	pMTL-YN3 in DH5α	(3)
1576	pDSW1728	Tetracycline-inducible *mCherry*	(6)
1628	pMTL-YN3-Δ*IVA*	pMTL-YN3-Δ*spoIVA* in HB101/pRK24	This study
1662	pMTL-YN1C	pMTL-YN1C in HB101/pRK24	(3)
1684	pMTL-YN1C-*IVA*	pMTL-YN1C-*spoIVA* in HB101/pRK24	This study
1756	pMTL-YN1C-*IVA* N491G	pMTL-YN1C-*spoIVA* N491G in HB101/pRK24	This study
1768	pMTL-YN1C-*mCherry-IVA*	pMTL-YN1C-*mCherry spoIVA* in HB101/pRK24	This study
1770	pJS107/*CD25622*	pJS107-*CD25622* targeting 38 bp in HB101/pRK24	This study
1817	pMTL-YN1C-*CD25622-VM*	pMTL-YN1C-*CD25622-VM* in HB101/pRK24	This study

Table S2. Primers used in this study.

Primer	Name	Sequence
532	3' Universal EBS	CGAAATTAGAAACTTGCGTTCAGTAAAC
1086	5’ NdeI *spoVM*	AGAAGACATATGAAAGTAGTAACAATAAAATTACCAAAATGG
1087	3’ XhoI *spoVM*	AGAAGACATATGAAAGTAGTAACAATAAAATTACCAAAATGG
1094	3’ HindIII IBS *spoVM* 32	AAAAAGCTTATAATTATCCTTAATTACCAAAATGGTGCGCCCAGATAGGGTG
1095	5’ BsrGI EBS1∂ *spoVM* 32	CAGATTGTACAAATGTGGTGATAACAGATAAGTCAAAATGGATAACTTACCTTTCTTTGT
1096	5’ EBS2 *spoVM* 32	TGAACGCAAGTTTCTAATTTCGGTTGTAATCCGATAGAGGAAAGTGTCT
1235	3’ *spoVM* sequencing 102 bp	GCGATTATAGATGGAGCTCC
1237	3’ XhoI *spoIVA*+TAA *subtilis*	AAATCTCGAGTTACAGGATGATGGCGATTAAGCCGC
1318	5’ NheI HA *spoIVA subtilis*	ATGGCTAGCTACCCGTACGACGTCCCGGATTATGCCTTGGAAAAGGTCGATATTTTCAAG
1609	3’ SalI *spoVM* no stop	AATGTCGACATCATTTTTACTCTTTCTCATAAATTTTAGG
1743	5’ Δ*spoIVA* flanking 1109 bp	GATTGTAAGGTAATAGTTTTAGCAGTTCC
1744	3’ Δ*spoIVA* flanking 1039 bp	CTAGATGTTTCTAATACCATATATTTGG
1746	5’ Δ*spoIVA* SOE	GGAGGAATAATATGAATAATAACATATACGTAAATGAAGGAAGTTCTAATATTATAAC
1747	3’ Δ*spoIVA* rev	GTTATAATATTAGAACTTCCTTCATTTACGTATATGTTATTATTCATATTATTCCTCC
2036	5’ NotI *spoIVA* Gibson	ttagggatgtaataagcggccgcCAATTAGCATTGTAGTTTACTAGTTTTTGTATATAGG
2037	3’ XhoI *spoIVA* Gibson	caagcttgcatgtctgcaggcctcgagCTTTGAAACAATCCTGTCGAAACATATAC
2148	5’ NotI *CD25622* Gibson	ggaattagggatgtaataagcggccgcGGTCTAAAATGGAAAACTGTCGC
2149	3’ XhoI *spoVM* Gibson	gccaagcttgcatgtctgcaggcctcgagGTTTAAAAGCCTACTTCCGTAAGTAGG
2177	5’ *spoIVA* N491G SOE	CAAAAAATTGTAAATGAAGGAAGTTCTGGTATTATAACTATTTTGTTATAAAAATATAGC
2178	3’ *spoIVA* N491G rev eos	GCTATATTTTTATAACAAAATAGTTATAATACCAGAACTTCCTTCATTTACAATTTTTTG
2186	5’ IBS1 *CD25622* 38	AAAAAAGCTTATAATTATCCTTAGTTGCCAGACAAGTGCGCCCAGATAGGGTG
2187	3’ EBS1∂ *CD25622* 38	CAGATTGTACAAATGTGGTGATAACAGATAAGTCAGACAAAGTAACTTACCTTTCTTTGT
2188	5’ EBS2 *CD225622* 38	TGAACGCAAGTTTCTAATTTCGATTGCAACTCGATAGAGGAAAGTGTCT
2202	5’ P*spoIVA-mCherry* SOE	CCCTTAACTCTAAATATAATTTAATAAGATTAGATGGTATCTAAAGGAGAAGAAGATAAT
2203	3’ P*spoIVA-mCherry* rev eos	ATTATCTTCTTCTCCTTTAGATACCATCTAATCTTATTAAATTATATTTAGAGTTAAGGG
2204	5’ *mCherry-spoIVA* SOE	GGAGGTATGGATGAATTATATAAAGTCGACATGTATAGGAGGAATAATATGAATAATAAC
2205	5’ *mCherry-spoIVA* rev eos	GTTATTATTCATATTATTCCTCCTATACATGTCGACTTTATATAATTCATCCATACCTCC
2272	5’ AscI Δ*spoIVA* Gibson-YN3	tcaattgttcaaaaaaataatggcGGCGCGCCCCATTTATAAAAGAAGGACAAGTTATTG
2273	3’ SbfI Δ*spoIVA* Gibson	agcaaggcaagaccgatcgggcccCCTGCAGGGACCTTTTATTTCATCACTAAATACACC

Restriction sites are underlined.

Table S3. Expression levels of select sporulation genes as measured by RNA-Seq.

Name	Spo0A			σ^F			σ^E			*spoIIID*$^-$			σ^K		
	BM	log_2 FC	adj P	BM	log_2 FC	adj P	BM	log_2 FC	adj P	BM	log_2 FC	adj P	BM	log_2 FC	adj P
spoVM	11	-5.0	$2.8x10^{-5}$	13	-2.4	0.01	13	-3.0	0.006	24	0.26	0.67	20	0.003	1
spoIVA	1147	-10.2	$2.7x10^{-51}$	1278	-3.8	$1.4x10^{-18}$	1266	-8.6	$9.7x10^{-53}$	1971	-0.59	0.09	1558	-1.3	0.26
sipL	655	-6.4	$2.1x10^{-45}$	746	-3.2	$2.8x10^{-18}$	730	-5.5	$2.7x10^{-47}$	1218	-0.28	0.42	902	-1.2	0.37

BM (base mean) represents the mean of read counts after they were divided by size factors to adjust for different sequencing depths. This value is the mean of the sample relative to wild type. *log_2FC* denotes log_2fold-change. A negative value indicates that the gene was reduced in expression relative to wild type. adj P represents that adjusted p-value associated with the fold change determined. The RNA-Seq data in the indicated strains has been previously published (7).

Table S4. Sporulation efficiency as measured by chloroform resistance.

**Strain**	**Sporulation efficiency**
630Δ*erm*	1 ± 0.34
Δ*spo0A*	$<10^{-6}$
VM$^-$	**0.21 ± 0.15**
25622$^-$	**0.18 ± 0.05**
VM$^-$/C	1.2 ± 1.0
25622$^-$/C	1.3 ± 1.1

Statistical significance relative to wild type 630Δ*erm* based on a minimum of three biological replicates was determined using an unpaired t-test of the *VM* mutant strains relative to wild type. Bolded text represents $p < 0.005$. Analyzing the data using a one-way ANOVA and Tukey's test did not detect a statistically significant difference between the *VM* mutant strains and wild type.

Table S5. Frequency of forespore morphological abnormalities detected by phase contrast microscopy.

	Free spores	Sporulating cells	Phase-bright forespores	Phase-dark forespores	Bearding	Misshapen forespores
WT	23% (95)	77% (316)	56% (178)	44% (138)	4% (13)	1% (4)
VM-	17% (74)	83% (349)	32% (113)	68% (236)	15% (52)	13% (44)
CD25622-	33% (178)	67% (359)	15% (55)	85% (304)	14% (50)	12% (43)
VM-C	17% (74)	83% (358)	46% (163)	54% (195)	4% (13)	2% (8)
CD25622-C	38% (257)	62% (426)	52% (222)	48% (204)	8% (32)	4% (17)

The percentage of free spores was determined by dividing the number of free spores (shown in parentheses) by the sum of free spores and sporulating cells (shown in parentheses) counted; the percentage of sporulating cells was determined accordingly. Sporulating cells include cells with phase-dark and phase-bright forespores but do not include cells that have completed asymmetric division, since these are indistinguishable from vegetative cells using phase-contrast microscopy. The percentage of phase-bright and phase-dark forespores, phase-dark extensions (bearding), and misshapen forespores was determined by dividing the number of cells with this phenotype (shown in parentheses) by the number of sporulating cells.

Supplemental References

1. Dineen SS, Villapakkam AC, Nordman JT, Sonenshein AL. 2007. Repression of *Clostridium difficil*e toxin gene expression by CodY. Mol Microbiol 66:206-19.
2. Ng YK, Ehsaan M, Philip S, Collery MM, Janoir C, Collignon A, Cartman ST, Minton NP. 2013. Expanding the repertoire of gene tools for precise manipulation of the *Clostridium difficile* genome: allelic exchange using *pyrE* alleles. PLoS One 8:e56051.
3. Donnelly ML, Li W, Li YQ, Hinkel L, Setlow P, Shen A. 2017. A *Clostridium difficile*-Specific, Gel-Forming Protein Required for Optimal Spore Germination. MBio 8.
4. Shen A, Lupardus PJ, Morell M, Ponder EL, Sadaghiani AM, Garcia KC, Bogyo M. 2009. Simplified, enhanced protein purification using an inducible, autoprocessing enzyme tag. PLoS One 4:e8119.
5. Putnam EE, Nock AM, Lawley TD, Shen A. 2013. SpoIVA and SipL are *Clostridium difficile* spore morphogenetic proteins. J Bacteriol 195:1214-25.
6. Ransom EM, Ellermeier CD, Weiss DS. 2015. Use of mCherry Red fluorescent protein for studies of protein localization and gene expression in *Clostridium difficile*. Appl Environ Microbiol 81:1652-60.
7. Pishdadian K, Fimlaid KA, Shen A. 2015. SpoIIID-mediated regulation of sigma(K) function during *Clostridium difficile* sporulation. Mol Microbiol 95:189-208.
